# A pharmacokinetic study and critical reappraisal of curcumin formulations enhancing bioavailability

**DOI:** 10.1016/j.isci.2025.112575

**Published:** 2025-05-03

**Authors:** Maurice A.G.M. Kroon, Hanneke W.M. van Laarhoven, Eleonora L. Swart, Olaf van Tellingen, E. Marleen Kemper

**Affiliations:** 1 Department of Pharmacy and Clinical Pharmacology, Amsterdam University Medical Center, AMC, 1105AZ Amsterdam, the Netherlands; 2 Department of Medical Oncology, Amsterdam University Medical Center, University of Amsterdam, 1081HV Amsterdam, the Netherlands; 3 Division of Pharmacology, The Netherlands Cancer Institute - Antoni van Leeuwenhoek Hospital, 1066CX Amsterdam, the Netherlands; 4 Department of Experimental Vascular Medicine, Amsterdam University Medical Center, AMC, 1105AZ Amsterdam, the Netherlands; 5 Cancer Center Amsterdam, Treatment and Quality of Life, 1081HV Amsterdam, the Netherlands

**Keywords:** Pharmaceutical science, Cell biology

## Abstract

This independent crossover study assessed curcumin bioavailability and excretion in nine healthy males receiving three formulations (AOV, Longvida, and NovaSOL) at about 570 mg, while AOV was also tested at 2280 mg, with and without piperine. Plasma levels of unconjugated curcumin remained below 2 nM in most cases, including high-dose AOV and piperine combinations. NovaSOL achieved the highest levels (6.7–38 nM at 30 min), but these rapidly declined and were still 100-fold lower than concentrations used *in vitro* to show biological effects. Curcumin conjugates exceeded 10 nM with all formulations, particularly NovaSOL, which showed 100-fold higher levels. Fecal recovery mainly included unconjugated curcuminoids and was high, except for NovaSOL, suggesting better intestinal absorption. However, even when using a formulation with enhanced uptake, plasma levels of unconjugated curcumin remained minimal. Piperine addition provided no benefit. The findings underscore that bioavailability claims should be based on unconjugated curcumin and not on its poorly membrane permeable conjugates.

## Highlights

• Unconjugated curcumin plasma levels remain low even with high doses
• NovaSOL achieves highest curcumin levels but still below required *in vitro* effects
• Piperine does not enhance curcumin bioavailability or systemic uptake
• Bioavailability claims should focus on unconjugated curcumin, not conjugates

## Graphical abstract

**Figure:**
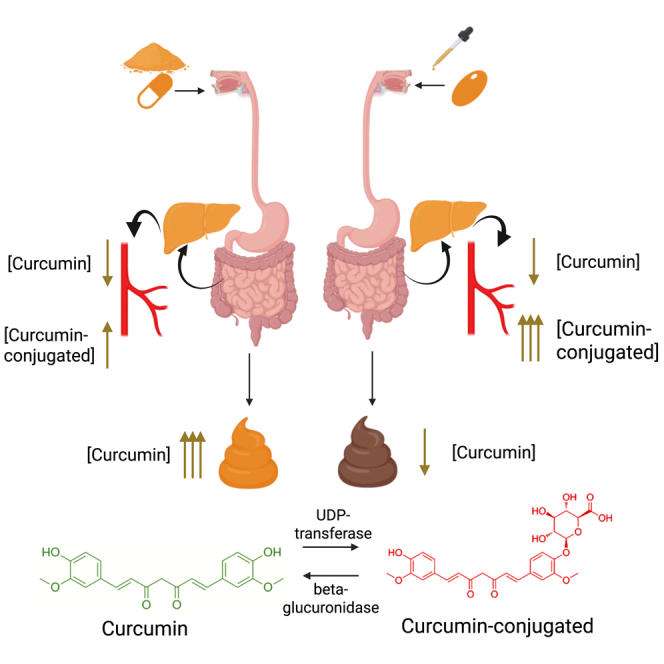

## Introduction

Due to the widespread health claims associated with curcumin, many individuals with cancer or other diseases, and even healthy individuals, resort to self-medication with curcumin as a proactive approach to manage their health. Curcumin has been extensively studied *in vitro* and in pre-clinical studies, exploring its therapeutic potential in areas such as oncology, inflammatory diseases, immunomodulation, diabetes, and Alzheimer’s disease.^1^^,^^2^^,^^3^ Based on these results, numerous clinical studies have been conducted and/or are ongoing. However, the promising results from *in vitro* and animal studies cannot consistently be replicated in clinical trials.^4^^,^^5^^,^^6^ This lack of efficacy is likely attributed to the low systemic bioavailability of curcumin after oral intake, which may stem from low aqueous solubility, rapid degradation in aqueous milieu, rapid and extensive first-pass metabolism, and efflux by p-glycoprotein (P-gp).^7^ Notably, a report combining results of many studies encompassing 309 cell lines, shows that the anticancer effects of unconjugated curcumin *in vitro* requires continued presence of concentrations ranging from 1 to 100 μM.^7^ Similarly high curcumin concentrations were required for effects on cardiac or microglial cells.^8^ However, unconjugated curcumin plasma levels typically remain much lower.^9^ Notably, *in vitro* active levels may even underestimate the required *in vivo* active plasma levels, as the fraction of unbound drug is lower in plasma compared to medium containing only 10% fetal calf serum. Besides these issues on low oral bioavailability, some of the positive preclinical results may actually be due to artifacts. Curcumin has been classified as a pan-assay interference compound, being agents that can produce false-positive results by interfering with the assay readout rather than through specific target interactions.^10^

Supplement companies have entered this market, offering various innovative and often expensive formulations with claims of improved bioavailability, such as “more, better, faster”, “proven higher bioavailability”, “185x greater bioavailability”, or “reaches plasma at least 65 times better than generic curcumin”.^11^^,^^12^^,^^13^ These claims are based on pharmacokinetic studies, authored and/or financially sponsored by the same companies.^11^^,^^14^^,^^15^^,^^16^^,^^17^^,^^18^ The formulations include nanoparticle delivery systems, oil-based products, a fenugreek dietary fiber formulation, and liquid capsules containing a micellar formulations, all designed to enhance the solubility and intestinal uptake of curcumin. Some formulations incorporate additives like piperine, intended to inhibit ABCB1-mediated efflux for improving intestinal absorption and/or to inhibit metabolic enzymes like CYP450 and UDP-glucuronosyltransferases (UGT) for reducing conversion of curcumin into more polar, hydrophilic conjugate metabolites.^19^ The addition of 20 mg piperine to a 2-g curcumin dosage was claimed to provide a 2000% increase in the bioavailability of curcumin in healthy volunteers.^20^ Importantly, all pharmacokinetic reports focus on total curcumin plasma levels, as sample pretreatment involves hydrolysis of conjugates using β-glucuronidase. These conjugated curcuminoid concentrations are present in higher concentrations; however, these polar conjugates are unable to cross cell membranes and enter cells, which would be required to exert pharmacologic activity, as shown in several *in vitro* studies investigating anti-proliferative effects on various cell lines (KBM-5, Jurkat, U266 and HepG2).^21^^,^^22^ Notably, in most cases, phase II metabolism is regarded as an inactivating process, increasing water solubility to enhance urinary excretion. Therefore, measuring the total of conjugated and unconjugated curcumin is not the correct metric to assess oral bioavailability and emphasis should be on unconjugated curcumin.^18^

The objective of this study is to investigate systemic exposure to unconjugated curcumin following oral administration, comparing different commercially available curcumin formulations. The curcumin formulations were selected based on the claims made by the manufacturers (i.e., 185x and 65x more absorption) and the most commonly used formulation from previous studies which are also readily available in Dutch supplemental stores.^23^ We measured concentrations of unconjugated curcumin, demethoxycurcumin (DMC), bisdemethoxycurcumin (BMC), and tetrahydrocurcumin (THC) in plasma, following supervised oral intake and serial blood sampling. Furthermore, we determined total (unconjugated + conjugated) curcumin plasma levels and the recovery of unconjugated and total curcumin in urine and feces collected over 24 and 48 h, respectively. The study’s design enables the examination of curcumin exposure in a controlled setting with healthy volunteers who do not have underlying gastrointestinal issues that might negatively influence absorption, distribution, metabolism, and excretion of curcumin. Participants have followed a strict diet in which no other intake of curcumin or piperine was allowed, enabling an accurate and unbiased comparison of the investigated curcumin formulations.

## Results

### Participants characteristics and clinical chemistry parameters

**Figure 1:**
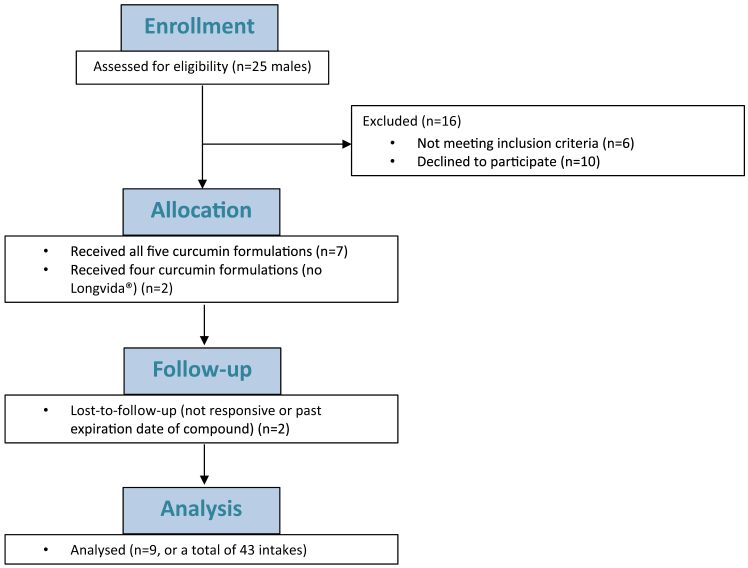
CONSERT flowchart of enrollment, allocation, follow up, and analysis Nine male participants (aged >18 years) were included of which 7 participants received 5 formulations and 2 participants received 4 formulations, resulting in 43 visit intakes.

A total of nine male participants were enrolled in this study between April 2018 and February 2022. During the COVID-19 pandemic, trials with healthy volunteers were halted, which led to a longer inclusion period. Among these nine participants, seven received all five different supplements, and two participants received only four different supplements due to loss to follow-up (see Figure 1). The study started in April 2018 with curcumin AOV801 (600 mg), AOV801 (2400 mg), curcumin AOV811 (2400 mg plus piperine 20 mg), and NovaSOL. An amendment was approved by the Ethics Committee of the Amsterdam UMC, location Amsterdam Medical Center (AMC), on July 29, 2019 (ref. 2017_079#B2019535) to include Longvida in this study. Participants that had already completed the study were asked to participate again for a single Longvida intake. Two participants did not reply leading to the loss to follow-up. This resulted in the analysis of seven out of nine participants for Longvida. The median age was 45; range 27–66 years. The mean weight was 86.5 kg ± 16.6 kg (SD), and the mean height was 178.6 cm ± 7.8 cm (SD).

Several clinical chemical parameters were assessed to investigate the impact of a single dose of curcumin. These results are detailed in Table S1. The table presents baseline values before ingestion of each specific curcumin formulation (see Table S1). All clinical chemistry parameters related to liver and kidney function, lipid profile, and electrolytes remained within the healthy reference range.

### Curcumin preparations contain less than indicated quantities

Each curcumin product was analyzed with our HPLC-MS/MS method to verify the exact amount of curcumin, DMC, and BMC per capsule.^24^ We tested three capsules of each supplement from two different batches in duplicate, except for Longvida where we verified one batch. Notably, none of the capsules contained the claimed curcumin/curcuminoid content (Table 1). Overall, the ratio of curcumin:DMC:BMC in the various supplements was consistently 80%:18%:2%. The actual measured curcumin amounts were used in our fecal and urinary recovery calculations described in the subsequent sections below.

**Table 1:** Content analysis of curcuminoids/curcumin formulations

Formulation	Curcuminoids			Curcumin		
	Claimed (mg)	Actual (mg)	bias %	Claimed (mg)	Actual (mg)	bias %
Curcumin 600 mg	570	386 ± 24	−32%	432	307.0 ± 17.7	−29%
Curcumin 600/5 mg	570	387 ± 8.5	−32%	432	307.4 ± 11.7	−28%
NovaSOL®	48	39.6 ± 1.5	−17%	36	31.4 ± 1.1	−13%
Longvida®	105	86.7 ± 5.1	−17%	105	70.5 ± 3.9	−33%

Curcuminoids include curcumin, DMC and BMC.

### Plasma levels of unconjugated curcumin are low with all formulations

**Figure 2:**
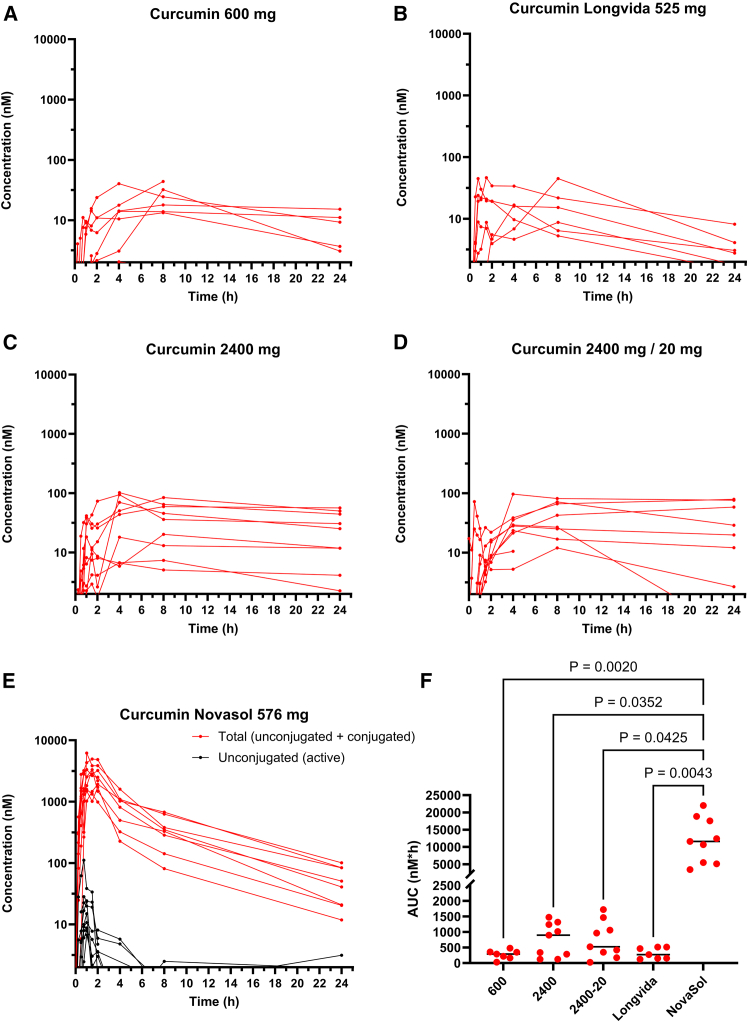
Plasma concentrations of curcumin Overview of plasma concentration – time curves of total (unconjugated + conjugated) and unconjugated curcumin of all participants following (A) 600 mg or (B) Longvida, (C) 2400 mg curcumin C3 complex, (D) 2400 mg curcumin C3 complex plus piperine 20 mg or (E) Novasol. Concentrations below the limit of quantitation (2.0 nM) are not shown. (F) displays the AUC_plasma_ of total curcumin. Statistical testing was done using the Kruskal-Wallis test.

Without β-glucuronidase pre-processing, thus measuring only unconjugated curcuminoids, the plasma levels of curcumin, DMC, and BMC were below the limit of quantitation (LOQ = 2 nM) in all participants receiving curcumin AOV801 (600 mg) (*n* = 9) (see Figure 2A), AOV801 (2400 mg) (*n* = 9) (see Figure 2B), AOV811 (2400 mg + piperine) (*n* = 9) (see Figure 2C), and Longvida (*n* = 7) (see Figure 2D) (see Table 2). With NovaSOL, all participants (*n* = 9) had measurable levels of unconjugated curcumin, with a median C_max_ of 14.9 nM (range: 6.7–38.4). Notably, the C_max_ was reached at 1 h, and rapidly declined to a median level of 1.2 nM (range: <LOQ – 5.7) after 4 h (see Figure 2E).

**Table 2:** Plasma pharmacokinetics of the different curcumin formulations

Formulation	Curcumin	AUC (nM.h) median (range)	C_max_ (nM) Median (range)	T_max_ (h)
Curcumin 600 mg	Unconjugated	n.d.	<LOQ	n.r.∗
	Total	289 (30–481)	21 (13–44)	8
Curcumin 2400 mg	Unconjugated	n.d.	<LOQ	n.r.∗
	Total	897 (122–1475)	44 (5.9–102)	4
Curcumin 2400 mg + piperine 20 mg	Unconjugated	n.d.	<LOQ	n.r.∗
	Total	524 (26–1724)	35 (12–81)	8
Longvida® (625 mg curcumin)	Unconjugated	n.d.	<LOQ	n.r.∗
	Total	271 (124–517)	15 (5.3–45)	8
NovaSOL® (576 mg curcuminoids)	Unconjugated	32 (13–94)	15 (6.7–38)	1
	Total	11590 (3450–22040)	2780 (1010–4950)	1.5

All samples were analyzed without and with pre-processing with β-glucuronidase to assess the levels of unconjugated and total (conjugated and unconjugated) curcumin in plasma.

n.d., not detected; ∗n.r., not reached; Total, unconjugated plus conjugated curcumin; LOQ, Lowest Limit of Quantification.

Following β-glucuronidase pre-processing, curcumin was detected in plasma of all participants, implying that orally administered curcumin reaches the systemic circulation predominantly as conjugate. Following curcumin AOV801 600 mg and AOV801 2400 mg formulations, the total curcumin median peak plasma levels were 21.2 and 43.7 nM, respectively (see Figures 2A and 2B; Table 2). In line with this, the AUC_(0–24 h)_ was higher, albeit not significantly and not proportionally (see Figure 2F). The T_max_ of curcumin conjugates for these formulations ranges between 4 and 8 h (see Figure 2), and a significant level relative to the peak plasma level persists even after 24 h, indicating continued absorption. Concomitant piperine to 2400 mg of curcumin did not accelerate or increase the systemic uptake and the AUC_plasma_ is similar to 2400 mg curcumin without piperine. The Longvida formulation resulted in an AUC, which was not significantly better than Curcumin 600 mg (Figure 2F; Table 2). NovaSOL intake resulted in the highest median total curcumin peak plasma concentrations of 2780 nM (range: 1010–4950 nM) (see Table 2). The C_max_ was reached at 1.5 h and from 4 h onwards, the plasma level declined according to first-order kinetics (half-life of about 3 h) suggesting that most absorption is taking place within the first 4 h. The AUC of curcumin with NovaSOL was significantly higher than that of all other formulations (see Figure 2F).

The other curcuminoids that are also present in these preparations (DMC and BMC) follow the same pattern as curcumin (see Figures S1 and S2). They are only present as conjugates and follow similar plasma concentration-time profiles as curcumin conjugates, albeit at proportionally lower levels. This also includes THC, an active metabolite from curcumin formed in the liver (see Figure S3).^25^ With NovaSOL intake, the highest median plasma levels and AUC_(0–24 h)_ (16860; range: 7890–37500 nM h) of THC were found, albeit also only in conjugated form (see Figure S3).

### Urinary excretion of curcuminoids is minor

**Figure 3:**
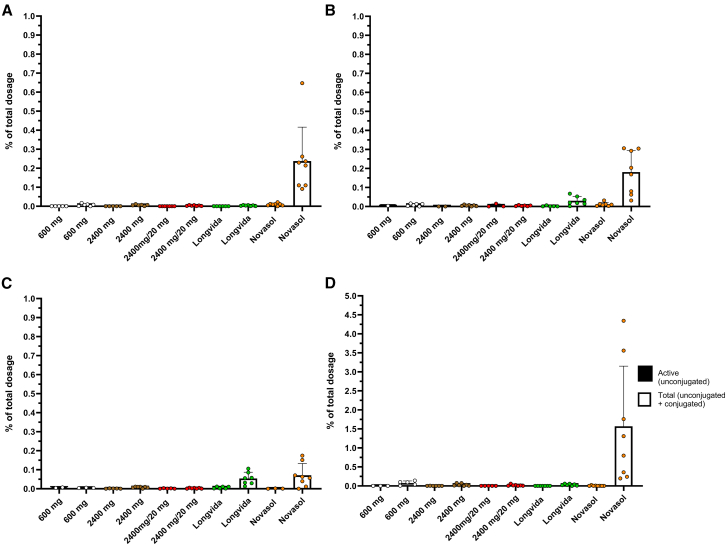
Urinary excretion of curcuminoids Overview of the recovery percentage (%) of the total dosage of (A) curcumin, (B) demethoxycurcumin, (C) bisdemethoxycurcumin, and (D) tetrahydrocurcumin in urine following the five different curcumin formulations. Each sample was pre-treated with (total curcumin) and without β-glucuronidase (unconjugated curcumin). Data are represented as mean ± SD.

As mentioned above, the actual curcuminoids contents of the curcumin preparation contain substantially less ingredients as indicated on the label. We used the actual quantities to calculate urinary and fecal excretion. In line with the higher conjugated curcumin plasma levels, the urinary excretion of conjugated curcumin following dosing of NovaSOL was significantly higher compared to all other curcumin formulations (See Figure 3A). Yet, the median recovery of total curcumin in urine with NovaSOL was only about 0.2% of the administered dose and the fraction of unconjugated curcumin herein was minor. Similar results were observed for the DMC and BMC after dosing of NovaSOL (See Figures 3B and 3C). The median urinary excretion of the THC following NovaSOL was 1.1% (range: 0.2–4.3) of the curcumin dose (See Figure 3D), which was for more than 99% conjugated. The urinary excretion of THC with all other curcumin formulations was substantially less.

### The predominant form of curcuminoids recovered in feces is typically unconjugated

**Figure 4:**
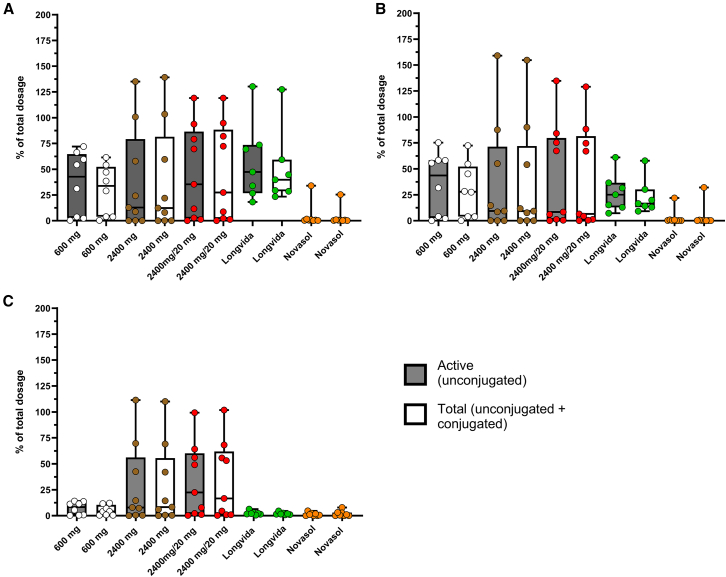
Fecal excretion of curcuminoids Overview of the recovery percentage (%) of the total dosage of (A) curcumin, (B) demethoxycurcumin, and (C) bisdemethoxycurcumin in feces following the five different curcumin formulations. Each sample was pre-treated with (total curcumin) and without β-glucuronidase (unconjugated curcumin). Data are represented as median and min. to max. (box and whiskers).

All feces produced per individual within 0–48 h was pooled and used to quantify the curcuminoids levels. In general, defecation occurred infrequently and in varying volumes. In total 71 defecations were collected with on average 1.6 defecations per supplement per participant. The most defecations (*n* = 40) were collected on t = 24 h visit. A total of 14 defecations were collected in the first 12 h with 17 defecations collected on the t = 48 h visit. Feces weights ranged from 98 to 1174 g. Per gram feces, 1 mL of 2% (w/v) bovine serum albumin (BSA) in water was used to homogenize each sample. Compared to the plasma and urine, the concentrations of unconjugated curcumin, DMC, and BMC were high in most cases (see Figure 4). Moreover, pre-processing samples with β-glucuronidase did not liberate more curcuminoids, indicating that the major fraction of curcuminoids recovered in feces is unconjugated. Overall, the recovered dose in feces was similar for all formulations (range median 13–47%), except for NovaSOL (median 0.6%). Excretion of the transformation product THC via the feces was negligible with all formulations. Notably, there was considerable variation between subjects (See Figure 4A). and this variation was observed with all curcuminoids (See Figures 4B and 4C). Some of this variation might be explained by incomplete feces sampling. Except for one patient who produced a single large stool within 12 h after intake and no more for the remaining 36 h, the fecal recovery using NovaSOL was consistently below 1.6% of the oral dose. This finding indicates that this lipid formulation indeed successfully improves absorption in the gut lumen. This result also suggests that most of the curcumin recovered in the feces has not been absorbed by the intestines during intestinal transit. Although intra-intestinal or even *ex vivo* deconjugation by bacterial glucuronidase of curcumin conjugates would be possible,^26^ the low fecal recovery of curcumin with NovaSOL indicates that hepatobiliary excretion of curcumin or curcumin conjugates is minimal. Otherwise, if a substantial portion of absorbed NovaSOL curcumin was excreted via bile as conjugates and then deconjugated by bacteria, the regenerated curcumin would lack the micelle formulation that facilitates efficient absorption. This fraction would likely be excreted in feces, similar to curcumin from other formulations. However, fecal recovery of curcumin from NovaSOL is low.

## Discussion

The claims of high and/or improved oral bioavailability of curcumin formulations are misleading, because they emphasize plasma levels of cell membrane impermeable curcumin conjugates. More importantly, the levels of unconjugated curcuminoids in plasma, which are expected to be the primarily pharmacologically active ingredients, typically remain significantly below the levels necessary to show effects in preclinical *in vitro* studies (1–100 μM).^1^^,^^7^^,^^9^ In fact, this work shows that strategies to improve the oral bioavailability of unconjugated curcumin by improving intestinal uptake are conceptually flawed.

The latter is clearly illustrated by NovaSOL, which comprises curcumin in a lipid/micellar vehicle within capsules to enhance dissolution and gastro-intestinal absorption. We have also investigated the fecal excretion of curcumin and observed near-complete intestinal absorption of curcumin formulated as NovaSOL. Notably, with NovaSOL, the plasma levels of total curcumin increase rapidly, reaching maximum levels within 2 h. Subsequently, total curcumin levels decline according to first-order elimination kinetics, with a half-life of approximately 3 h. These plasma concentration–time profiles of total curcumin indicate rapid, nearly complete uptake from the gut within 4 h. A similar plasma concentration–time profile is observed for total THC. Thus, while the NovaSOL formulation effectively facilitates rapid and complete uptake from the gastrointestinal tract, the rate of first-pass biotransformation of curcumin outpaces the rate of uptake, limiting the oral bioavailability of unconjugated, curcumin. The AUC_(0–24 h)_ of total curcumin was approximately 12-fold higher than reported by Fanca-Berthon et al., being proportional to the 12-fold higher dose level.^16^ However, the unconjugated curcumin AUC_(0–24 h)_ was similar, despite the 12-fold higher dose, indicating that higher doses do not necessarily result in higher unconjugated curcumin levels. Similarly, the increase of the AUC_(0–24 h)_ of total THC was less than 12-fold in our series, which may suggest saturation of the enzymes involved in reduction of curcumin to THC.^27^ The finding that a very minor fraction of curcumin enters the systemic circulation as unconjugated compound, even with NovaSOL is in line with previous studies.^11^^,^^16^^,^^28^^,^^29^ Other studies only focus on total curcumin levels.^29^^,^^30^ Hence, in order to promote their products over other curcumin formulations, these manufacturers have come up with alternative reasoning why their product would be better. For example, a study conducted by Naturex SA (France)^16^ compared their TurmiPure Gold formulation (TPG), with NovaSOL, a phytosome formulation (PHYT) from Meriva^28^ and standard turmeric extracts, both without (STE) and with piperine (TEP). Instead of comparing these products at similar dose levels, they used the dosages for each formulation as recommended by the respective manufacturer. Next, dose-normalized AUCs were used to calculate relative bioavailabilities, and to claim that TPG has a 342-fold higher relative bioavailability (0–8 h) of unconjugated curcumin. Intriguingly, the AUC_(0–8 h)_ of TPG (containing 74.3 mg curcumin) versus STE (1425 mg) was 33.6 and 30.6 ng h/ml, respectively. Indeed, this would imply a higher relative oral bioavailability, albeit only 20-fold and not 342-fold. More importantly, however, all formulations (TPG, NovaSOL, PHYT, STE, or TEP) applied at their recommended dosages resulted in plasma levels of unconjugated curcumin below 20 ng/mL (50 nM). As outlined above, we administered NovaSOL at a 12-fold higher dose, yet observed similar C_max_ and/or AUC_(0–24 h)_ of unconjugated curcumin (Table 2). Next to this, we have shown that a 4-fold increase in dosage (AOV801 2400 mg vs. AOV801 600 mg) does not lead to notably higher unconjugated curcumin plasma concentrations. Furthermore, the addition of piperine to curcumin does not increase unconjugated plasma levels or even total plasma levels. This inability of piperine to enhance systemic bioavailability has also been demonstrated by others,^15^^,^^25^ but has received little attention. Based on a single study by Shoba et al.,^20^ piperine has become a commonly used additive. However, our results and those of others^15^^,^^25^ clearly demonstrate that addition of piperine is useless. Although this is an inter-study comparison, these findings suggest that first-pass conjugation of curcumin is very effective and seems to cap unconjugated curcumin plasma levels.

Whereas other naturally occurring curcuminoids (DMC and BMC), or metabolites like THC may accumulate in cells *in vitro* and exert pharmacological activity alike curcumin, sulfate and glucuronide conjugates do not, as has been shown in several papers.^21^^,^^22^ This observation is consistent with the understanding that these more hydrophilic conjugates have a reduced ability to permeate cellular membranes. Alternatively, it has been suggested that high levels of conjugated curcumin in blood may still pose biological relevance through site-specific deconjugation. Kunihiro et al.^31^ provided evidence that β-glucuronidase expressed by hematopoietic cells can cause locally elevated levels of unconjugated curcumin that exerted pharmacological activity on osteoclastogenesis. Alternatively, Ozawa et al.^32^ postulate that β-glucuronidase would support the maintenance of high levels of free curcumin in blood. They further demonstrate antitumor efficacy of curcumin-glucuronide against a HCT116 tumor model in mice. Referring to this work, Fanca-Berthon et al. emphasizes that future studies should not be limited to unconjugated curcumin.^16^ However, while site-specific expression of β-glucuronidase may have local effects on unconjugated curcumin bioavailability, the likelihood that this has clinical relevance is questionable. Notably, the effects reported by Kunihiro et al.^31^ were observed following extremely high dose levels (500 mg/kg) of curcumin, resulting in curcumin-glucuronide plasma levels in mice of 100 μM (370 μg/mL). Similarly, Ozawa et al. applied i.v. injections of curcumin monoglucuronide (CMG) resulting in peak plasma levels of CMG higher than 100 μM.^32^ Intriguingly, already at 1 min after administration, the level of unconjugated curcumin peaked at 10 μM and then declined at a similar rate as CMG. This challenges the notion that β-glucuronidase is involved in this conversion. Notably, the study does not report on the purity of the CMG, in particular the presence of curcumin in the CMG preparation administered to the animals. Even if glucuronide conjugates may pose pharmacological activity via local deconjugation, their clinical relevance is likely negligible. Even NovaSOL, administered at 12 times the manufacturers recommended dose, yielded peak plasma levels of curcumin-conjugates below 10 μM. This is still at least 10 times lower than the levels achieved in the aforementioned studies in mice.

Interestingly, our study shows that the concentration of unconjugated curcumin in feces is very high. Since this is most likely a reflection of high intra-intestinal exposure, local effects of curcumin inside the intestinal lumen may occur. A systematic review including 6 randomized, placebo-controlled studies did show that various curcumin formulations in combination with mesalamine (5-ASA) maintained remission in patients with colitis ulcerosa.^33^ Several other randomized controlled trials found that curcumin might possibly induce clinical and histological remission when given in combination with mesalamine.^34^^,^^35^^,^^36^^,^^37^ Alterations in the gut microbiota might have effects on short chain fatty acids produced by several bacterial strains, which could be an explanation for effects on glucose and serum free fatty acids in randomized studies.^38^^,^^39^ Notably, high gastrointestinal curcumin concentrations may already be achieved with inexpensive formulations of curcumin, while expensive micellar-based formulations with rapid intestinal uptake may even work counterproductive.

In summary, none of the tested “enhanced” formulations achieved plasma levels of unconjugated curcumin that even remotely approached the levels necessary to trigger a pharmacodynamic response *in vitro*. Although formulations like NovaSOL showed rapid and complete uptake from the gastrointestinal tract, resulting in higher plasma levels of conjugated curcumin, the plasma concentrations of unconjugated curcumin remained about 1,000 times lower than preclinical thresholds required for activity. Moreover, adding piperine does not enhance the systemic exposure to curcumin, both unconjugated and total. Claims of enhanced bioavailability based solely on total curcumin levels overstate the actual benefits of the product. Scientific journals should critically evaluate manuscripts and advise authors against making unwarranted assertions. This is particularly important as supplement manufacturers often use these claims in marketing, presenting products as “Scientifically Proven” or “More, Better, Faster”. These assertions risk misleading consumers who may lack scientific expertise to critically assess their validity.

### Limitations of the study

A limitation of this study concerns the fact that the correct collection and storage conditions of feces relied on self-reporting by the study participants. Moreover, the amount of stool and the time of defecation was highly variable. Especially when only a single stool sample was produced relatively soon after intake, this might have resulted in incomplete sampling. More controlled and extensive fecal sampling could have mitigated this issue, reducing variability in the results.

We included only five different formulations in this study, so caution is needed when extrapolating the findings to other formulations. However, we anticipate that the conclusion—that merely increasing intestinal uptake does not address the issue of low systemic bioavailability of unconjugated curcumin—applies broadly to all curcumin formulations. Furthermore, another limitation is the food intake after t = 4 h. No food was provided beyond this time point, and although participants were instructed not to eat until after t = 8 h, they were unsupervised during this period. Additionally, while participants were instructed to arrive fasting at t = 24 h, no monitoring occurred after t = 8 h and the start of the fasting period 4 h before t = 24 h. This may lead to minor variations in the measured data; however, no significant impact is expected.

Lastly, curcumin concentrations that demonstrate effectiveness in *in vitro* studies cannot be directly extrapolated as a threshold for pharmacologically active plasma concentrations. However, it is evident that there is a substantial gap between *in vitro* and *in vivo* concentrations.

## Resource availability

### Lead contact

Further information can be directed to the lead contact: Olaf van Tellingen (o.v.tellingen@nki.nl).

### Materials availability

This study did not generate new unique reagents.

### Data and code availability

• All data associated in this study are presented in this paper or in supplemental information.
• The paper does not report original code.
• All raw data required to reanalyze the data reported in this paper is available from the lead contact upon request.

## Acknowledgments

This study was funded by an internal grant of the Pharmacy department of the Amsterdam UMC, location 10.13039/501100003180Academic Medical Center.

## Author contributions

Conceptualization, M.A.G.M.K., O.V.T., and E.M.K.; methodology, M.A.G.M.K., O.V.T., and E.M.K.; formal analysis, M.A.G.M.K., O.V.T., and E.M.K.; investigation, M.A.G.M.K.; resources, M.A.G.M.K. and O.V.T.; writing – original draft, M.A.G.M.K.; writing – review and editing, M.A.G.M.K., H.W.M.V.L., E.L.S., O.V.T., and E.M.K.; visualization, M.A.G.M.K., O.V.T., and E.M.K.; supervision, H.W.M.V.L., E.L.S., O.V.T., and E.M.K.; project administration, M.A.G.M.K., O.V.T., and E.M.K.; funding acquisition, E.M.K.

## Declaration of interests

The authors declare no competing interests.

## Declaration of generative AI and AI-assisted technologies in the writing process

During the preparation of this work the author(s) used ChatGPT to scan for grammatical errors. After using this tool/service, the authors reviewed and edited the content as needed and take full responsibility for the content of the publication.

## STAR★Methods

### Key resources table

**Table:** 

REAGENT or RESOURCE	SOURCE	IDENTIFIER
**Biological samples**		

Human blood, urine and fecal samples	This study	N/A
Omniplasma	Sanquin	https://prothya.com/product/omniplasma/

**Chemicals, peptides, and recombinant proteins**		

Bovine Serum Albumin (BSA)	Sigma Aldrich, Schnelldorf, Germany	CAS 9048-46-8
AOV801 Curcumin 600 mg capsules	MCO Health B.V., Almere, The Netherlands	https://www.aov.nl/
AOV811 Curcumin 600 mg plus piperine 5 mg capsules	MCO Health B.V., Almere, The Netherlands	https://www.aov.nl/
NovaSOL®	AQUANOVA AG, Darmstadt, Germany	https://aquanova.de/
Longvida®	Verdure Sciences, Noblesville, U.S.A.	https://vs-corp.com/longvida/
Dimethyl sulfoxide	Sigma Aldrich, Schnelldorf, Germany	CAS 67-68-5
Methanol ≥99.9%, MS	Sigma Aldrich, Schnelldorf, Germany	CAS 67-56-1
β-glucoronidase from Helix Pomatia, Type H-1	Sigma Aldrich, Schnelldorf, Germany	CAS 9001-45-0
*tert*-butyl methyl ether (TBME)	Sigma Aldrich, Schnelldorf, Germany	CAS 1634-04-4
curcumin (≥98% HPLC)	Sigma-Aldrich, St. Louis, MOI, USA	CAS 458-37-7
Demethoxycurcumin (≥98% HPLC)	Sigma-Aldrich, St. Louis, MOI, USA	CAS 22608-11-3
Bisdemethoxycurcumin (≥98% HPLC)	Sigma-Aldrich, St. Louis, MOI, USA	CAS 33171-05-0
Piperine (analytical standard)	Sigma-Aldrich, St. Louis, MOI, USA	CAS 94-62-2
Tetrahydrocurcumin (≥98% HPLC)	Toronto Research Chemicals (Toronto, Canada)	CAS 3606-04-1
Curcumin-D6	Toronto Research Chemicals (Toronto, Canada)	CAS 1246833-26-0
(2E)-demethoxycurcumin-d7	Toronto Research Chemicals (Toronto, Canada)	CAS 22608-11-3
(E,E)Bisdemethoxycurcumin-D8	Toronto Research Chemicals (Toronto, Canada)	CAS 33171-05-0
Tetrahydrocurcumin-D6	Toronto Research Chemicals (Toronto, Canada)	CAS 1794898-13-7
Piperine-D10	Toronto Research Chemicals (Toronto, Canada)	CAS 94-62-2

**Software and algorithms**		

GraphPad Prism, version 10.2.0	GraphPad Software, La Jolla California, USA	https://www.graphpad.com
Analyst®, versions 1.6.2 and 1.7.2	Sciex, Framingham, MA, USA	https://sciex.com/

**Other**		

JumboMix 3500 P CC	Interscience, Saint Nom la Bretèche, France	https://www.interscience.com/
API 4000 triple mass spectrometer	Sciex, Framingham, MA, USA	https://sciex.com/
UltiMate 3000 LC System	Dionex, Sunnyvale, CA, USA	https://www.thermofisher.com/
3.5 micron ZORBAX Extend-C18 particles	Agilent, Santa Clara, CA, USA	https://www.agilent.com/
4.0 × 2.0 mm Securityguard C18 pre-column	Phenomex, Utrecht, The Netherlands	https://www.phenomenex.com/

### Experimental model and study participants details

#### Study design, participants and curcumin products

The study protocol was reviewed and approved by the Ethics Committee of the Amsterdam UMC, Academic Medical Center, The Netherlands (approval 2017_079#B2017314). The study was registered in the Dutch Clinical Trial Register with ID NL6552. This is a single-center, open-label, cross-over intervention study involving nine healthy participants. Participants underwent screening within 21 days before dosing on day 1 and were included if they were male, aged ≥18 years, generally considered healthy, able to provide written informed consent, willing to follow the dietary regimen, and able to complete the entire study. Exclusion criteria were any major illness in the past 3 months, gastrointestinal conditions that might affect supplement absorption, a history of cholecystectomy or other bile duct abnormalities, metabolic or endocrine diseases, active or former drug abuse or alcoholism (>3 units of alcohol per day), use of prescription or non-prescription drugs and herbal or dietary supplements in the past 30 days (other than drugs used for their local effect without influencing curcumin pharmacokinetics), use of tobacco products, consumption of high amounts of curcumin and black pepper in daily food/beverages, known intolerance to curcumin or black pepper, participated in another clinical trial in the 3 months before the study start, consumption of alcohol or caffeine products within 3 days before each study day, strenuous exercise for at least 3 days before each study day (defined as over 1 h of exercise per day), or consumption of grapefruit and grapefruit-containing products or star fruit within 3 days before each study day. After screening and meeting the inclusion criteria, each subject received a curcumin product on the day of visit. A minimum wash-out period of five days was implemented during the cross-over period (due to the estimated half-life of curcumin metabolites being around 8.8–13 h).^7^^,^^40^ All participants adhered to dietary restrictions for three days before the start of the study including not consuming foods with curcumin (e.g., Indian Curry) and piperine (black pepper). A list of foods lacking these substances was provided. Each participant recorded their dietary intake during the days using a paper diary and/or an online dietary system.

Seven participants were of White Dutch ethnicity, one participant was of Pakistan ethnicity and one participant was of Curaçao ethnicity. All participants provided informed consent during the screening visit. On each visit day, participants received one of the following commercial products in a cross-over setting: curcumin-C3 complex 600 mg (AOV, MCO Health B.V., Almere, The Netherlands), curcumin-C3 complex 2400 mg (4 capsules of curcumin-C3 complex 600 mg) (AOV, MCO Health B.V., Almere, The Netherlands), curcumin-C3 complex with piperine 2400 mg/20 mg (4 capsules of 600 mg curcumin-C3 complex with 5 mg piperine) (AOV, MCO Health B.V., Almere, The Netherlands), curcumin complex formulated in polysorbate 80 (576 mg curcuminoids, 12 capsules, each capsule contains 48 mg curcuminoids) (NovaSOL, AQUANOVA AG, Darmstadt, Germany), and curcumin in Solid Lipid Curcumin Particles (SLCP) Technology formulation (625 mg curcuminoids, 5 capsules, each capsules contains 125 mg curcuminoids) (Longvida, Verdure Sciences, Noblesville, U.S.A.). In general, extracts from the root of Curcuma Longa contain multiple curcuminoids, with curcumin (±70%) being most abundant, followed by DMC (±21%) and BMC (±9%).^41^ These three curcuminoids are collectively known as the C3 complex. The recommended dosages by the manufacturers are one capsules twice a day for the AOV products and one capsule once a day for the NovaSOL and Longvida products. NovaSOL contains a claimed 48 mg of curcuminoids, whereas Longvida claims 500 mg of curcuminoids (standardized to 21% curcumin or 105 mg). To compare the four formulations, NovaSOL was administered as a single dose of 12 capsules, totaling a dosage of 576 mg of curcuminoids. Longvida was given as a single dose of 5 capsules, totaling a dosage of 525 mg of curcumin. The AOV products contain 600 mg of curcumin extract (95% curcuminoids equaling to 570 mg curcuminoids). All 9 participants, except 2, received all formulations. The two exceptions received all formulations, except Longvida, for reasons explained in the main text.

### Method details

#### Study procedures

Participants were instructed to visit the AMC fasted (fasted for at least 4 h before t = 0) in the morning of day 1 (around 8:00 a.m.). They were allowed to consume water. Two hours after the curcumin intake, foods and beverages were provided by the investigators to prevent consumption of products containing curcumin/piperine. Blood samples were collected at t = 0 (h), t = 0.25 (h), t = 0.5 (h), t = 0.75 (h), t = 1 (h), t = 1.5 (h), t = 2 (h), t = 4 (h), t = 8 (h), and t = 24 (h) after intake. For the final blood and urine sample participants returned to the site at the t = 0 (h) the next day. Urine was collected from t = 0 (h) until 24 h after intake, and feces were collected until 48 h after intake. Participants were instructed to keep their urine refrigerated during the 24 h of collecting, while immediately freezing any obtained fecal sample. When a subject did not have any defecation within this time frame, the first post-intake defecation was collected. All feces collected within this 48-h period were combined in one bag and homogenized (JumboMix 3500 P CC, Interscience, Saint Nom la Bretèche, France) with 2% (w/v) Bovine Serum Albumin (BSA) in water using a 1:1 (w/v) ratio for 20–30 min with increasing strokes/second to a maximum of 6 strokes/second and decreasing pedal distance to ensure thorough mixing until homogeneous. After homogenization, aliquots of the feces samples (together with plasma and urine samples) were stored at −20°C until further analysis.

The following pharmacokinetic parameters were determined: Plasma area under the curve (AUC_plasma_), plasma maximum concentration (C_max_), time of C_max_ (T_max_), mean and standard deviation (SD) of urine and feces concentration. The AUC_plasma_ was calculated using GraphPad Prism version 10.2.0 from each plasma concentration curve. Standard laboratory testing were performed on liver and renal function, osmolality, glucose and lipid metabolism, and anti-inflammatory markers by the Central Clinical Chemistry Laboratory of the Amsterdam UMC, location AMC, The Netherlands: sodium, potassium, calcium, phosphate, chloride, urea, creatinine, alanine aminotransferase (ALAT), aspartate aminotransferase (ASAT), gamma-GT, osmolality, IgM, alkaline phosphatase, albumin, lactate dehydrogenase, C-reactive protein (CRP), glucose, and parameters related to glucose, lipid metabolism, and inflammation. Baseline physical measurements included participants’ height (cm) and weight (kg).

#### Processing samples with the enzyme β-glucuronidase

Upon entering the body via the intestines, curcuminoids undergo rapid conversion into inactive hydrophilic glucuronide and sulfate conjugates. Most HPLC-MS/MS-based analytical methods detect only unconjugated curcuminoids. To measure conjugated metabolites, samples were pre-incubated with β-glucuronidase to deconjugate the curcuminoids, before further sample pretreatment. Each sample was divided into two parts: one part was treated with β-glucuronidase, while the other part was not. Analysis of both samples provides levels of total (conjugated and unconjugated) and active (unconjugated) compound in the sample.^42^

#### Analysis of curcumin content in various supplements

Each supplement was tested on individual curcuminoid content to calculate the bias to the claimed content by the manufacturer. From each batch, three capsules, within the expiration date and stored in the original container at room temperature protected from light exposure, were dissolved in 50 mL DMSO per capsule using a volumetric flask. Each capsule was further diluted in MeOH:H_2_O 60:40 until within the expected range of 100–400 nM. A total of 50 μL of sample was analyzed together with calibration samples ranging from 2 to 400 nM and internal standard mix (see paragraph below for exact composition), prepared in MeOH: H_2_O 60:40 followed with the HPLC-MS/MS analysis described below.^24^

#### Analysis of curcumin, demethoxycurcumin, bisdemethoxycurcumin, tetrahydrocurcumin and piperine in plasma, urine and fecal samples

Plasma, urine, and feces samples were analyzed using a validated HPLC-MS/MS method.^24^ On the day of analysis, samples were thawed and pretreated with and without β-glucoronidase from *Helix Pomatia*, Type H-1 (Sigma Aldrich, Schnelldorf, Germany), which contains ≥300,000 U/g β-glucoronidase and ≥10,000 U/g sulfatase. The samples pretreated with β-glucuronidase were incubated with 20 U/μL β-glucuronidase (pH = 5.0) at 37°C and 850 revolutions per minute (RPM) for 1 h. Previous test showed no further conversion of conjugated forms after 1 h at 37°C and 850 rpm, yielding about 92% conversion. Liquid-liquid extraction with *tert*-butyl methyl ether (TBME) was used as the organic solvent to extract the compounds from the plasma, urine, or fecal samples. The reference standards curcumin (≥98% HPLC), DMC (≥98% HPLC), BMC (≥98% HPLC) and piperine analytical standard were purchased from Sigma-Aldrich (St. Louis, MOI, USA), The Netherlands). THC (≥98% HPLC) and the Internal Standards (IS) curcumin-D6, (2E)-demethoxycurcumin-d7, (E,E)Bisdemethoxycurcumin-D8, Tetrahydrocurcumin-D6 and Piperine-D10 were purchased from Toronto Research Chemicals (Toronto, Canada). All compounds were quantified in a single analytical run. The LC-MS/MS system consisted of an API 4000 triple mass spectrometer (Sciex, Framingham, MA, USA) with an electrospray ionization source coupled to an UltiMate 3000 LC System (Dionex, Sunnyvale, CA). Samples were separated using a 100 × 3 mm column packed with 3.5 micron ZORBAX Extend-C18 particles (Agilent, Santa Clara, CA, USA), preceded by a 4.0 × 2.0 mm Securityguard C18 pre-column (Phenomenex, Utrecht, The Netherlands). Separation was done at ambient temperature using a mixture of mobile phase A (0.1% formic acid in Milli-Q water (v/v)) and mobile phase B (MeOH) in a 3 min gradient from 50% to 95%B, followed by 95%B that was maintained for 6 min and then 6 min re-equilibrated at 20%B. The flow rate was 0.20 mL/min and the total run time 18 min per sample. System control and data analysis was done using Analyst 1.5.1 software (Sciex). During sample preparation and HPLC-MS/MS analysis, light exposure was minimized by working in dim light and away from direct sunlight, covering the samples with aluminum foil as much as possible, and keeping the lid of the autosampler tray closed. Each analytical run included a set of freshly prepared calibration samples containing all compounds in the validated range of 2 nM–400 nM. Concentrations higher than 400 nM were diluted to fit within the validated range.

### Quantification and statistical analysis

#### Sample size calculation and statistical analysis

The sample size calculation is based on the fact that, with a sample size of 9, a single-group repeated measures analysis of variance with alpha = 0.05 (without adjustment for multiple comparisons) will have 98% power to detect a difference in the means in the efficacy parameter (AUC) of curcumin, characterized by an effect size of 0.8889 (e.g., a Variance of means of 2.00, a standard deviation at each level of 1.5, and a between-level correlation of 0.0). Parameters will be compared using unpaired T-tests or nonparametric tests, depending on result distribution, or two-way ANOVA calculation with Dunnett’s multiple comparison test. Differences in AUC among different preparations will be evaluated using Kruskal-Wallis tests in Graphpad Prism (version 10.2.0).

### Additional resources

The study was registered in the Dutch Clinical Trial Register with ID NL6552.

## References

[1] Aggarwal B.B., Sung B. (2009). Pharmacological basis for the role of curcumin in chronic diseases: an age-old spice with modern targets. Trends Pharmacol. Sci..

[2] Epstein J., Sanderson I.R., Macdonald T.T. (2010). Curcumin as a therapeutic agent: the evidence from in vitro, animal and human studies. Br. J. Nutr..

[3] Jayaprakasha G.K., Jagan Mohan Rao L., Sakariah K.K. (2005). Chemistry and biological activities of C. longa. Trends Food Sci. Technol..

[4] Mazzanti G., Di Giacomo S. (2016). Curcumin and Resveratrol in the Management of Cognitive Disorders: What is the Clinical Evidence?. Molecules.

[5] Liu S., Liu J., He L., Liu L., Cheng B., Zhou F., Cao D., He Y. (2022). A Comprehensive Review on the Benefits and Problems of Curcumin with Respect to Human Health. Molecules.

[6] Khosravi M.A., Seifert R. (2024). Clinical trials on curcumin in relation to its bioavailability and effect on malignant diseases: critical analysis. Naunyn-Schmiedebergs Arch Pharmacol.

[7] Heger M., van Golen R.F., Broekgaarden M., Michel M.C. (2014). The molecular basis for the pharmacokinetics and pharmacodynamics of curcumin and its metabolites in relation to cancer. Pharmacol. Rev..

[8] Brosková Z., Drábiková K., Sotníková R., Fialová S., Knezl V. (2013). Effect of plant polyphenols on ischemia-reperfusion injury of the isolated rat heart and vessels. Phytother. Res..

[9] Dytrych P., Kejík Z., Hajduch J., Kaplánek R., Veselá K., Kučnirová K., Skaličková M., Venhauerová A., Hoskovec D., Martásek P., Jakubek M. (2023). Therapeutic potential and limitations of curcumin as antimetastatic agent. Biomed. Pharmacother..

[10] Nelson K.M., Dahlin J.L., Bisson J., Graham J., Pauli G.F., Walters M.A. (2017). The Essential Medicinal Chemistry of Curcumin. J. Med. Chem..

[11] Schiborr C., Kocher A., Behnam D., Jandasek J., Toelstede S., Frank J. (2014). The oral bioavailability of curcumin from micronized powder and liquid micelles is significantly increased in healthy humans and differs between sexes. Mol. Nutr. Food Res..

[12] Gota V.S., Maru G.B., Soni T.G., Gandhi T.R., Kochar N., Agarwal M.G. (2010). Safety and Pharmacokinetics of a Solid Lipid Curcumin Particle Formulation in Osteosarcoma Patients and Healthy Volunteers. J. Agric. Food Chem..

[13] Jamwal R. (2018). Bioavailable curcumin formulations: A review of pharmacokinetic studies in healthy volunteers. J. Integr. Med..

[14] Kocher A., Schiborr C., Behnam D., Frank J. (2015). The oral biovailability of curcuminoids in healthy humans is markedly enhanced by micellar solubilisation but not further improved by simultaneous ingestion of sesamin, ferulic acid, naringenin and xanthohumol. J. Funct.Foods.

[15] Kanai M., Imaizumi A., Otsuka Y., Sasaki H., Hashiguchi M., Tsujiko K., Matsumoto S., Ishiguro H., Chiba T. (2012). Dose-escalation and pharmacokinetic study of nanoparticle curcumin, a potential anticancer agent with improved bioavailability, in healthy human volunteers. Cancer Chemother. Pharmacol..

[16] Fança-Berthon P., Tenon M., Bouter-Banon S.L., Manfré A., Maudet C., Dion A., Chevallier H., Laval J., van Breemen R.B. (2021). Pharmacokinetics of a Single Dose of Turmeric Curcuminoids Depends on Formulation: Results of a Human Crossover Study. J. Nutr..

[17] Pandaran Sudheeran S., Jacob D., Natinga Mulakal J., Gopinathan Nair G., Maliakel A., Maliakel B., Kuttan R., Im K. (2016). Safety, Tolerance, and Enhanced Efficacy of a Bioavailable Formulation of Curcumin With Fenugreek Dietary Fiber on Occupational Stress: A Randomized, Double-Blind, Placebo-Controlled Pilot Study. J. Clin. Psychopharmacol..

[18] Kumar D., Jacob D., Subash P.S., Maliakkal A., Johannah N.M., Kuttan R., Maliakel B., Konda V., Krishnakumar I.M. (2016). Enhanced bioavailability and relative distribution of free (unconjugated) curcuminoids following the oral administration of a food-grade formulation with fenugreek dietary fibre: A randomised double-blind crossover study. J. Funct.Foods.

[19] Bhardwaj R.K., Glaeser H., Becquemont L., Klotz U., Gupta S.K., Fromm M.F. (2002). Piperine, a major constituent of black pepper, inhibits human P-glycoprotein and CYP3A4. J. Pharmacol. Exp. Ther..

[20] Shoba G., Joy D., Joseph T., Majeed M., Rajendran R., Srinivas P.S. (1998). Influence of piperine on the pharmacokinetics of curcumin in animals and human volunteers. Planta Med..

[21] Pal A., Sung B., Bhanu Prasad B.A., Schuber P.T., Prasad S., Aggarwal B.B., Bornmann W.G. (2014). Curcumin glucuronides: assessing the proliferative activity against human cell lines. Bioorg. Med. Chem..

[22] Shoji M., Nakagawa K., Watanabe A., Tsuduki T., Yamada T., Kuwahara S., Kimura F., Miyazawa T. (2014). Comparison of the effects of curcumin and curcumin glucuronide in human hepatocellular carcinoma HepG2 cells. Food Chem..

[23] Kroon M.A.G.M., Berbee J.K., Majait S., Swart E.L., van Tellingen O., van Laarhoven H.W.M., Kemper E.M. (2023). Non-therapeutic plasma levels in individuals utilizing curcumin supplements in daily life. Front. Nutr..

[24] Kroon M.A.G.M., van Laarhoven H.W.M., Swart E.L., Kemper E.M., van Tellingen O. (2023). A validated HPLC-MS/MS method for simultaneously analyzing curcumin, demethoxycurcumin, bisdemethoxycurcumin, tetra-hydrocurcumin and piperine in human plasma, urine or feces. Heliyon.

[25] Zhou M., Li R., Hua H., Dai Y., Yin Z., Li L., Zeng J., Yang M., Zhao J., Tan R. (2024). The role of tetrahydrocurcumin in disease prevention and treatment. Food Funct..

[26] Dashnyam P., Mudududdla R., Hsieh T.J., Lin T.C., Lin H.Y., Chen P.Y., Hsu C.Y., Lin C.H. (2018). β-Glucuronidases of opportunistic bacteria are the major contributors to xenobiotic-induced toxicity in the gut. Sci. Rep..

[27] Hoehle S.I., Pfeiffer E., Sólyom A.M., Metzler M. (2006). Metabolism of curcuminoids in tissue slices and subcellular fractions from rat liver. J. Agric. Food Chem..

[28] Cuomo J., Appendino G., Dern A.S., Schneider E., McKinnon T.P., Brown M.J., Togni S., Dixon B.M. (2011). Comparative absorption of a standardized curcuminoid mixture and its lecithin formulation. J. Nat. Prod..

[29] Flory S., Sus N., Haas K., Jehle S., Kienhöfer E., Waehler R., Adler G., Venturelli S., Frank J. (2021). Increasing post-digestive solubility of curcumin is the most successful strategy to improve its oral bioavailability: A randomized cross-over trial in healthy adults and In Vitro bioaccessibility experiments. Mol. Nutr. Food Res..

[30] Jäger R., Lowery R.P., Calvanese A.V., Joy J.M., Purpura M., Wilson J.M. (2014). Comparative absorption of curcumin formulations. Nutrition J.

[31] Kunihiro A.G., Luis P.B., Brickey J.A., Frye J.B., Chow H.H.S., Schneider C., Funk J.L. (2019). Beta-Glucuronidase Catalyzes Deconjugation and Activation of Curcumin-Glucuronide in Bone. J. Nat. Prod..

[32] Ozawa H., Imaizumi A., Sumi Y., Hashimoto T., Kanai M., Makino Y., Tsuda T., Takahashi N., Kakeya H. (2017). Curcumin beta-D-Glucuronide Plays an Important Role to Keep High Levels of Free-Form Curcumin in the Blood. Biol. Pharm. Bull..

[33] Coelho M.R., Romi M.D., Ferreira D.M.T.P., Zaltman C., Soares-Mota M. (2020). The Use of Curcumin as a Complementary Therapy in Ulcerative Colitis: A Systematic Review of Randomized Controlled Clinical Trials. Nutrients.

[34] Kedia S., Bhatia V., Thareja S., Garg S., Mouli V.P., Bopanna S., Tiwari V., Makharia G., Ahuja V. (2017). Low dose oral curcumin is not effective in induction of remission in mild to moderate ulcerative colitis: Results from a randomized double blind placebo controlled trial. World J. Gastrointest. Pharmacol. Ther..

[35] Lang A., Salomon N., Wu J., Kopylov U., Lahat A., Har-noi O., Ching J., Cheong P.K., Avidan B., Gamus D. (2015). Curcumin add-on therapy for induction of remission in mild-moderate active Ulcerative Colitis: A multi-center, prospective, randomized, double-blind, placebo-controlled trial. J. Crohns Colitis..

[36] Sadeghi N., Mansoori A., Shayesteh A., Hashemi S.J. (2020). The effect of curcumin supplementation on clinical outcomes and inflammatory markers in patients with ulcerative colitis. Phytother. Res..

[37] Banerjee R., Pal P., Penmetsa A., Kathi P., Girish G., Goren I., Reddy D.N. (2021). Novel Bioenhanced Curcumin With Mesalamine for Induction of Clinical and Endoscopic Remission in Mild-to-Moderate Ulcerative Colitis: a Randomized Double-Blind Placebo-controlled Pilot Study. J. Clin. Gastroenterol..

[38] Jamilian M., Foroozanfard F., Kavossian E., Aghadavod E., Shafabakhsh R., Hoseini A., Asemi Z. (2020). Effects of curcumin on body weight, glycemic control and serum lipids in women with polycystic ovary syndrome: A randomized, double-blind, placebo-controlled trial. Clin. Nutr. ESPEN.

[39] Na L.X., Li Y., Pan H.Z., Zhou X.L., Sun D.J., Meng M., Li X.X., Sun C.H. (2013). Curcuminoids exert glucose-lowering effect in type 2 diabetes by decreasing serum free fatty acids: a double-blind, placebo-controlled trial. Mol. Nutr. Food Res..

[40] Vareed S.K., Kakarala M., Ruffin M.T., Crowell J.A., Normolle D.P., Djuric Z., Brenner D.E. (2008). Pharmacokinetics of curcumin conjugate metabolites in healthy human subjects. Cancer Epidemiol. Biomarkers Prev..

[41] Huang M.T., Ma W., Lu Y.P., Chang R.L., Fisher C., Manchand P.S., Newmark H.L., Conney A.H. (1995). Effects of curcumin, demethoxycurcumin, bisdemethoxycurcumin and tetrahydrocurcumin on 12-O-tetradecanoylphorbol-13-acetate-induced tumor promotion. Carcinogenesis.

[42] Awolade P., Cele N., Kerru N., Gummidi L., Oluwakemi E., Singh P. (2020). Therapeutic significance of β-glucuronidase activity and its inhibitors: A review. Eur. J. Med. Chem..

