# Supplementary material for: A pharmacokinetic study and critical reappraisal of curcumin formulations enhancing bioavailability
Source: iScience. 2025 May 3;28(6):112575. doi: 10.1016/j.isci.2025.112575 (PMC12144411; doi:10.1016/j.isci.2025.112575)
Supplement: Document S1. Figures S1–S3 and Table S1 [file mmc1.pdf]

iScience, Volume *28*

Supplemental information

A pharmacokinetic study and critical reappraisal of curcumin formulations enhancing bioavailability

Maurice A.G.M. Kroon, Hanneke W.M. van Laarhoven, Eleonora L. Swart, Olaf van Tellingen, and E. Marleen Kemper

SUPPLEMENTARY FIGURES AND TABLES

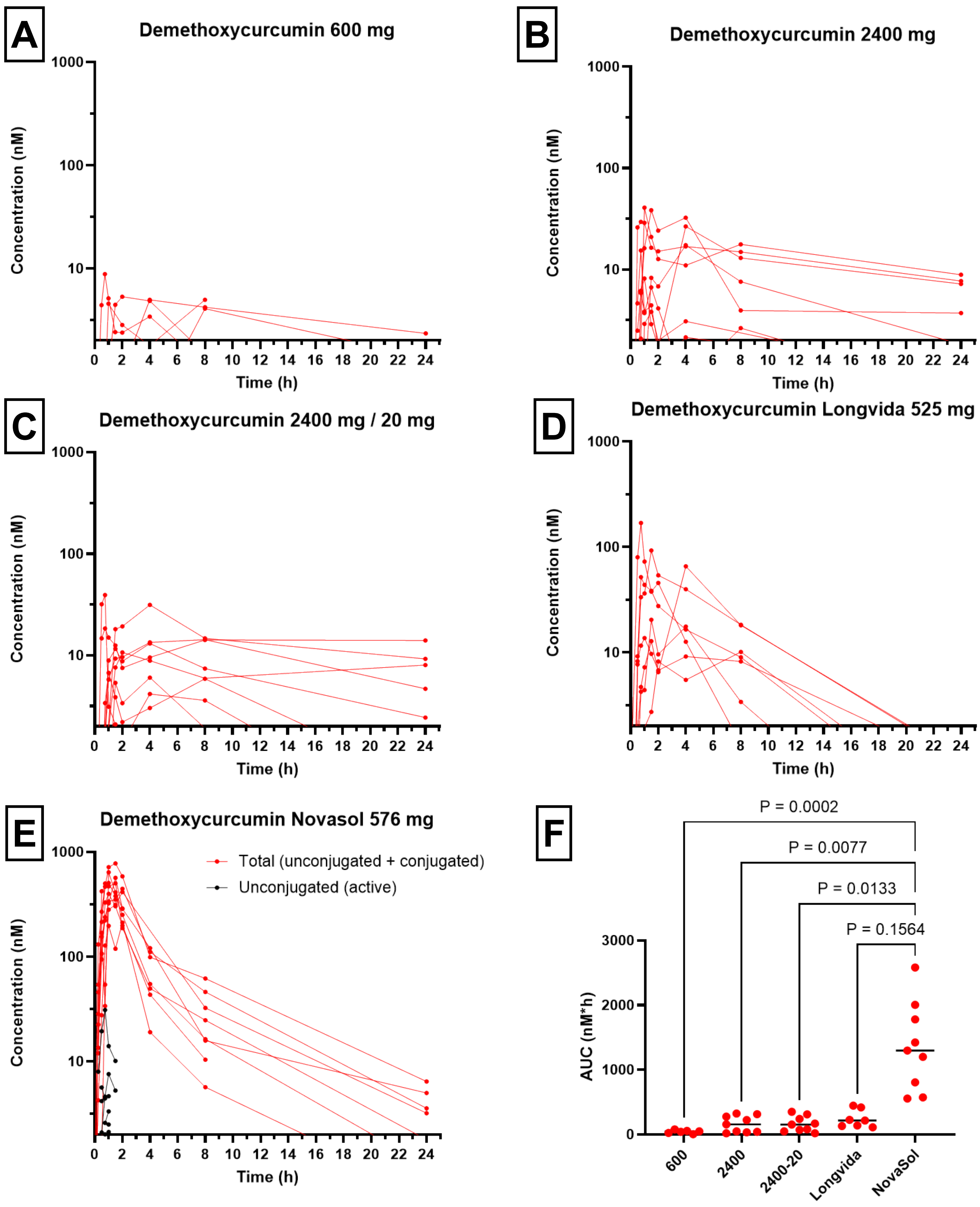

Figure S1. Plasma concentrations of demethoxycurcumin. Overview of plasma concentration – time curves of total (unconjugated + conjugated) and unconjugated (active) demethoxycurcumin of all participants following (A) 600 mg or (B) 2400 mg curcumin C3 complex, (C) 2400 mg curcumin C3 complex plus piperine 20 mg, (D) Longvida® or (E) NovaSOL®. Concentrations below the limit of quantitation (2.0 nM) are not shown. Panel F displays the AUC_{plasma} of total demethoxycurcumin. Statistical testing was done using the Kruskal-Wallis test.

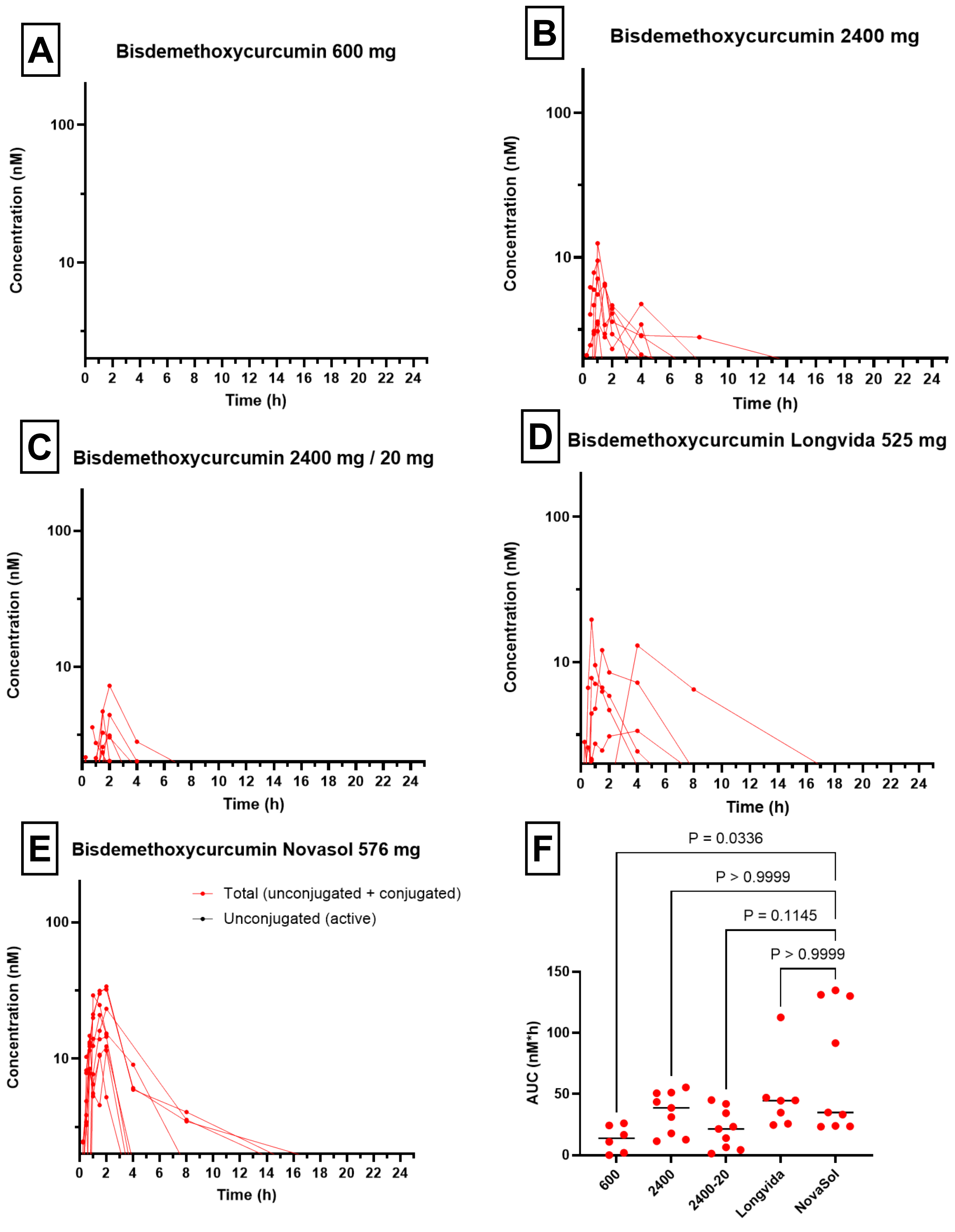

Figure S2. **Plasma concentrations of bisdemethoxycurcumin.** Overview of plasma concentration – time curves of total (unconjugated + conjugated) and unconjugated (active) bisdemethoxycurcumin of all participants following (A) 600 mg or (B) 2400 mg curcumin C3 complex, (C) 2400 mg curcumin C3 complex plus piperine 20 mg, (D) Longvida® or (E) NovaSOL®. Concentrations below the limit of quantitation (2.0 nM) are not shown. Panel F displays the AUC_{plasma} of total demethoxycurcumin. Statistical testing was done using the Kruskal-Wallis test.

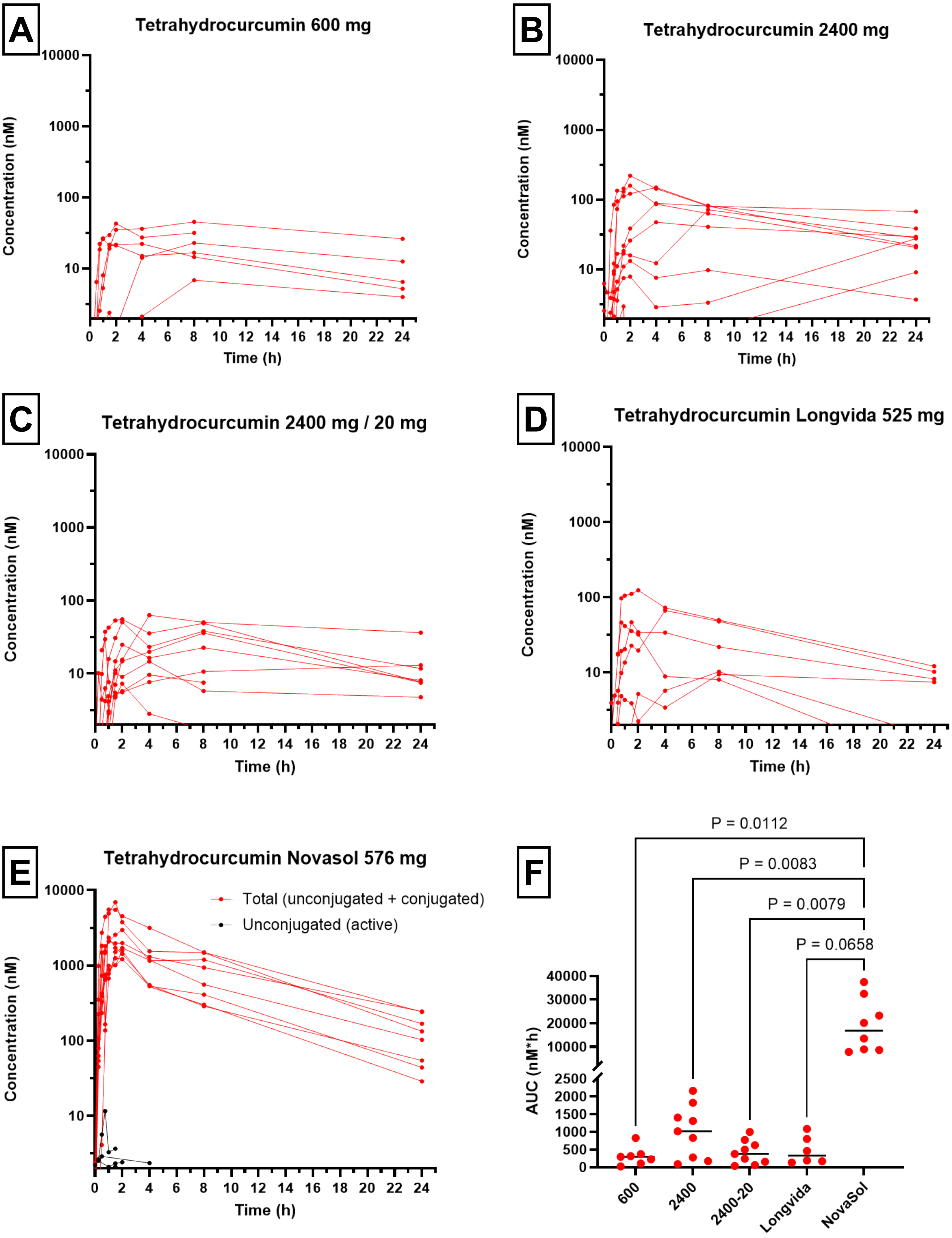

Figure S3. **Plasma concentrations of tetrahydrocurcumin**. Overview of plasma concentration – time curves of total (unconjugated + conjugated) and unconjugated (active) tetrahydrocurcumin of all participants following (A) 600 mg or (B) 2400 mg curcumin C3 complex, (C) 2400 mg curcumin C3 complex plus piperine 20 mg, (D) Longvida® or (E) NovaSOL®. Concentrations below the limit of quantitation (2.0 nM) are not shown. Panel F displays the AUC_{plasma} of total demethoxycurcumin. Statistical testing was done using the Kruskal-Wallis test.

	Reference limits	Curcumin 600 mg (mean ± SD) (n=9)	Curcumin 2400 mg (mean ± SD) (n=9)	Curcumin 2400 mg / 20 mg piperine (mean ± SD) (n=9)	Novasol® (mean ± SD) (n=9)	Longvida® (mean ± SD) (n=7)
		Baseline (t=0h)	Baseline (t=0h)	Baseline (t=0h)	Baseline (t=0h)	Baseline (t=0h)
Glucose (mmol/L)	4.1 - 5.6	4.96 ± 0.5	4.9 ± 0.3	5.2 ± 0.4	5.1 ± 0.3	5.2 ± 0.4
LDH (U/L 37C)	0 - 248	182 ± 40	171 ± 27	165 ± 28	113 ± 86	160 ± 18
Totale cholesterol (mmol/L)	<5.0	5.3 ± 0.7	5.3 ± 0.7	5.3 ± 0.7	5.1 ± 0.7	5.6 ± 1.2
HDL (mmol/L)	>1.0	1.2 ± 0.3	1.7 ± 1.2	1.2 ± 0.3	1.2 ± 0.3	1.2 ± 0.3
LDL-chol. (mmol/L)	1.61 - 3.37	3.5 ± 0.6	3.55 ± 0.6	3.6 ± 0.7	3.4 ± 0.7	3.7 ± 1.1
Triglyceride (mmol/L)	<2.0	1.0 ± 0.3	1.2 ± 0.7	1.3 ± 0.6	1.3 ± 0.6	1.4 ± 0.7
ASAT (U/L 37C)	0 - 40	23 ± 5.3	23 ± 4.9	22 ± 5.3	22 ± 4.3	23 ± 7.6
ALAT (U/L 37C)	0 - 45	29 ± 12	28 ± 6.4	25 ± 7.2	25 ± 4.7	31 ± 14
AP (U/L 37C)	40 - 120	72 ± 14	69 ± 22	65 ±22	62 ± 20	64 ± 26
Gamma-GT (U/L 37C)	0 - 60	19 ± 7.9	22 ± 8.4	22 ± 9.9	20 ± 11	31 ± 39
CRP (mg/L)	0 - 5	1.4 ± 1.1	1.2 ± 0.9	2.3 ± 3.3	1.8 ± 1.3	1.4 ± 1.2
Ureum (mmol/L)	2.1 - 7.1	6.1 ± 1.7	6.3 ± 2.3	6.0 ± 1.6	6.0 ± 1.1	5.8 ± 1.1
Creatinine (µmol/L)	75 - 110	78 ± 14	80 ± 14	81 ± 18	81 ± 14	76 ± 11
eGFR (mL/min/1.73 m^2)	> 60	89 ± 1.7	90 ± 1.0	88 ± 3.7	90 ± 0.0	90 ± 0.0
Albumin (g/L)	35 - 50	44 ± 2.9	44 ± 1.4	44 ± 2.6	43 ± 1.4	44 ± 1.6
Sodium (mmol/L)	135 - 145	139 ± 2.5	139 ± 1.8	139 ± 1.6	139 ± 1.3	138 ± 2.1
Potassium (mmol/L)	3.5 - 4.5	4.0 ± 0.4	3.9 ± 0.3	3.9 ± 0.3	4.1 ± 0.4	4.0 ± 0.4
Calcium (mmol/L)	2.2 - 2.6	2.3 ± 0.1	2.4 ± 0.1	2.4 ± 0.1	2.3 ±0.1	2.3 ± 0.1
Phosphate (mmol/L)	0.85 - 1.65	1.1 ± 0.2	1.1 ± 0.3	1.1 ± 0.2	1.0 ± 0.2	1.1 ± 0.1
Chloride (mmol/L)	98 - 107	104 ± 4.3	103 ± 3.1	103 ± 3.5	104 ± 2.6	101 ± 1.8

Supplemental Table S1. **Overview of the baseline clinical chemistry parameters**

Baseline parameters were assessed before oral intake of each curcumin formulation: 600 mg curcumin C3 complex, curcumin 2400 mg curcumin C3 complex, curcumin 2400 mg mg curcumin C3 complex plus added piperine 20 mg, Novasol® and Longvida®.
